# Editorial

**Published:** 1872-09

**Authors:** 


### Editorial.

***Sanitary Reform.***

The mortality lists for the month of July, 1872, present an aggregate of 1,372, or a daily average of 44¼ deaths. This frightful mortality has increased during the first week of August to about 60 per day, or an increase of 25 per cent. For the last five years, during which sanitary statistics have been recorded, the annual mortality has reached its maximum during the latter part of August and first of September. Should this rule apply, and the same rates of progression prevail, in this, as in former years, the maximum death rate for 1872 will be something truly terrible.

We have no intention to create a panic either at home or abroad, as there is no necessity for any such feeling, but only to urge upon the attention of the city government and of citizens generally, the absolute necessity for the organization and maintenance of a special Department for the preservation and protection of Public Health.

Some five years ago, indeed, an effort was made by certain public-spirited physicians to secure the legislative sanction necessary to the accomplishment of this object; but the effort was rendered abortive, and the legislation inefficient, through the machination of private interests, to which the law was perverted, in its very construction, and the public good made subservient.

The destruction of the city by the Great Fire has given the opportunity for the inauguration of municipal reform in several directions in which its necessity, long felt by the more intelligent, was thus clearly demonstrated to all. Let the opportunity be improved in this direction likewise. Let not our city, rising like a phœnix from its ashes, prove a Necropolis, its marble palaces magnificent tombs.

For proof of the necessity for sanitary reform, let physicians read the reports of the so-called Board of Health, which should no longer be suffered to constitute the standing jokes of newspaper reporters, but should be read as notes of warning, to all who have the future health and prosperity of the city at heart. We have been playing at sanitary reform long enough; five years of endurance, and over a quarter of a million of dollars for the performance, ought to satisfy its most enthusiastic admirer, with the length and cost of the farce.

Now, let us go to work in earnest, inaugurate sanitary reforms, based upon scientific principles, and approved by common sense and general experience—reforms which shall make themselves apparent to all; which shall have removed reeking heaps of garbage from our streets and alleys; abolished open privies, foci of typhoid fever, from back yards; broken up forever the infamous traffic in swill-milk, by which so many hundreds of children are annually sacrificed upon the altar of mammon; which shall have taught the people the rudiments, at least, of the laws of health, and compelled their observance; and shall have abolished moreover all contracts.

Let us at the close of another five years have completed a sanitary-topographical survey of the whole city, which shall exhibit the sanitary condition of every lot, and be capable of indicating all future changes thereupon; epidemiological charts, exhibiting the limits, and variations in the intensity of epidemic and endemic diseases; meteorological tables, indicating the relation of atmospheric influences to human health; ethnological charts and tables, showing the influence of nationality and national habits in the production and modification of disease. Let something be done which shall earn confidence at home and respect abroad. Let us have a mass of statistics to which medical men can refer for information in the elucidation of sanitary questions, with a feeling of security in their authenticity, accuracy, and good faith, instead of a collection of long-winded "reports" as useless practically, as they are unintelligible grammatically.

Let us, by studying the nature and character of the influences and agencies detrimental to public health, be able to counteract them, and be prepared to present statistics of a steadily decreasing, instead of an increasing, ratio of mortality.

There is scarcely an intelligent physician in the city, who does not recognize the necessity for a thorough change in the Health Department. Unfortunately, this can only be accomplished by a radical change in the law under which the Department was organized. The first step toward this end must be the rescinding of that legislative absurdity by which a member of an administrative body becomes his own executive officer. That this mischievous clause in the law has been the principal obstacle to its efficient execution is well known and distinctly avowed by members of the Board.

Inadequate as is the law, in relation to its proposed objects, it might still have been made to accomplish much good, in the hands of some of its administrators, who have throughout been possessed of intelligence and actuated by good faith, had not this insuperable barrier to the efficient execution of any law constantly interposed.

So long as this law remains unaltered, the Health Department of the city of Chicago will stand a tribute of legislative gullibility to personal trickery. H.

---

### *St. Joseph's Hospital.*

This hospital is situated in the North Division of the city, on the corner of Sophia and Burling streets, and under the control of the Sisters of Charity. It is a very large, well-ventilated building, perfect in its internal appointments, affording every facility to those wishing medical treatment—whether in the private rooms, or general wards—and has connected with it a "Visiting Staff" of physicians and surgeons of recognized talent and ability.

In the lecture room of this institution, a series of clinical lectures will be delivered to the students of medicine, illustrated by the various diseases in the general wards, and every care taken to make the instruction practical and complete.

On the 16th of Sept. prox., the regular meeting of the visiting staff will take place in their room at the hospital, when, among the business transacted, assignments to duty will be made, and a course of lectures agreed upon. The necessary information will be then furnished all colleges at the earliest practicable moment.

We give below the gentlemen comprising the "Medical Board" of St. Joseph's Hospital:

VISITING STAFF OF PHYSICIANS AND SURGEONS.

*Medical Department*—Professors J. P. Ross, J. H. Etheridge, E. L. Holmes, and F. B. Norcom, M.D.

*Obstetrical and Gynecological Department*—Professor De L. Miller, and A. H. Cook, M.D.

*Surgical Department*—W. D. Winer, M.D., and Profs. J. W. Freer, M. Gunn, and E. Powell.

STAFF OF EXTERNES.

O. J. H. Adams, M.D., and L. W. Case, M.D.

CONSULTING STAFF.

Prof. H. A. Johnson, and E. Schmidt, M.D.

J. W. Freer, *President pro tem.*

F. B. Norcom, *Sec. pro tem.*

---

### *Brainard Medical Society.*

The Society met in the rooms of the Young Men's Christian and Library Association, in Logansport, Indiana, June 27th, 1872.

Dr. L. D. Glazebrook, President elect, delivered his inaugural address, and took the chair.

Members present: L. D. Glazebrook, James Thomas, I. B. Washburn, W. S. Cleland, J. B. Moore, J. B. Hoag, H. Garner, G. W. Nafe, and W. H. Bell.

On motion, Drs. Wm. Lomax, Williams and Ayers, of Grant Co. Medical Society, Dr. Dickens, Wabash Co. Society, Dr. Ballou, White Co. Society, and Dr. Beckner, Newton Co. Society, were elected honorary members.

On motion, all the members of the profession present were invited to participate in the proceedings of the meeting. Those present were G. N. Fitch, A. Coleman, A. B. Buchanan, J. Herman, J. M. Justice, R. Faber, and J. H. Shultz, of Logansport, and J. A. Adrian, of Onward.

Dr. Hoag read an essay on the Duties of Physicians, which was discussed by Drs. Fitch, Ayers and Lomax.

Dr. Bell read a paper on Neuralgia of the Heart. It was discussed by Drs. Fitch and Hoag.

At this time a number of physicians arrived. They were J. A. Comingore, R. N. Todd, and Thad. M. Stevens, members of the faculty of the Indiana Medical College; Drs. R. Q. Wilson, E. A. Armstrong, I. C. Johnson, Wm. Scott, H. C. Cole, and W. K. Mavity, of Kokomo, members of the Howard Co. Society; Dr. Higgins, Peru, and Drs. J. A. Meek and O. C. Irwin, Bunker Hill, of the Miami Co. Society.

Dr. Washburn read a paper on *Epidemic Meningeal Typhus*, or Spotted Fever, which gave rise to a very lively discussion, participated in by Drs. Comingore, Fitch, Todd, Scott, Stevens, Cole, Bell, Cleland, Hoag, Wilson, Glazebrook, Thomas, and Washburn. The discussion revealed the fact that but few *post mortem* examinations had been made, and those were principally, if not all, of sporadic cases.

On motion, adjourned to meet in Miramac, Ind., Oct. 3, 1872.

I. B. Washburn, Sec.

